# Evaluation of the intention to use the electronic medical record (EMR) by health professionals in healthcare facilities of Libreville and Owendo in Gabon

**DOI:** 10.1093/jamiaopen/ooac096

**Published:** 2022-11-22

**Authors:** Gaëtan Moukoumbi Lipenguet, Edgard-Brice Ngoungou, Tamara Roberts, Euloge Ibinga, Prudence Amani Gnamien, Jean Engohang-Ndong, Jérôme Wittwer

**Affiliations:** EMOS Team—BPH U1219, University of Bordeaux, Bordeaux, France; Department of Epidemiology–Biostatistics and Medical Informatics (DEBIM), Faculty of Medicine, University of Health Sciences, Libreville, Gabon; Department of Epidemiology–Biostatistics and Medical Informatics (DEBIM), Faculty of Medicine, University of Health Sciences, Libreville, Gabon; Inserm U1094, IRD U270, Univ. Limoges, CHU Limoges, EpiMaCT—Epidemiology of Chronic Diseases in Tropical Zone, Institute of Epidemiology and Tropical Neurology, OmegaHealth, Limoges, France; Research Unit in Epidemiology of Chronic Diseases and Environmental Health (UREMCSE), University of Health Sciences, Libreville, Gabon; EMOS Team—BPH U1219, University of Bordeaux, Bordeaux, France; Department of Epidemiology–Biostatistics and Medical Informatics (DEBIM), Faculty of Medicine, University of Health Sciences, Libreville, Gabon; Research Unit in Epidemiology of Chronic Diseases and Environmental Health (UREMCSE), University of Health Sciences, Libreville, Gabon; EMOS Team—BPH U1219, University of Bordeaux, Bordeaux, France; Department of Epidemiology–Biostatistics and Medical Informatics (DEBIM), Faculty of Medicine, University of Health Sciences, Libreville, Gabon; Research Unit in Epidemiology of Chronic Diseases and Environmental Health (UREMCSE), University of Health Sciences, Libreville, Gabon; Department of Biological Sciences, Kent State University at Tuscarawas, New Philadelphia, Ohio, USA; EMOS Team—BPH U1219, University of Bordeaux, Bordeaux, France

**Keywords:** intention to use, electronic medical record, TAM, Gabon

## Abstract

**Introduction:**

Health systems in several countries have integrated information and communication technologies into their operations. Electronic medical records (EMRs) are at the core of patient care. The working of these EMRs requires their acceptance and use by medical and paramedical personnel. The objective of this study was to empirically evaluate the intention of health professionals to use these EMRs.

**Materials and Methods:**

A questionnaire on the intention of health professionals to use the EMR was developed following a Likert scale. The survey was done via in-person interviews of health professionals in major health facilities in the cities of Libreville and Owendo in Gabon. The technology acceptance model (TAM) was tested using a step-down logistic regression analysis to identify the main factors explaining the intention of health professionals to use the EMR.

**Results:**

A total of 218 health professionals responded to the questionnaire. Thirty-eight percent (38%) of respondents were male. The average age was 41.33 years (±8.98 years) and the average length of service at work in the system was 12.02 years (±8.47 years). The integrated model showed that the intention to use the EMR was significantly associated with the perceived usefulness, the subjective standard, and experience. No socio-demographic variables explained the intention to use the EMR.

**Conclusion:**

The perceived ease, familiarity with the computer, and motivation are not associated with the intention to use the EMR. Actions should be taken to raise awareness and train health professionals to motivate them to accept and use EMRs in their medical practices.

## INTRODUCTION

The quest for efficiency and effectiveness in the management of patient care leads healthcare facilities to deploy clinical information systems (CISs) and to implement electronic medical records (EMRs). An EMR is a private and secure record that collects data on the medical history of a patient.^1^ An EMR is also designed to promote the exchange of information on the care path of a patient between health professionals and/or between organizations.

In its quest to optimize the patient care path, the Ministry of Health of Gabon has set up a CIS to communicate EMRs created in healthcare facilities such as university hospital centers, polyclinics, and clinics. This innovation in the healthcare facilities of Gabon was made possible through funding received from the World Bank as part of the health information system improvement project. This reinforcement of health systems aims to digitize the paper-based systems in healthcare facilities lacking a digital information system and to establish interoperability between new and/or currently existing systems in some health structures. University hospital centers (Centres Hospitaliers Universitaires) and some healthcare structures have each a CIS. However, and unfortunately, these CIS do not communicate with each other. Thus, the project to strengthen the information system will allow these CISs to be interconnected in order to share patient information between health professionals through the EMR.

The effectiveness of EMRs is conditioned by the evolution of best professional practices of all categories of workers involved in the care of the patient. The efficiency sought by the deployment of the EMR entails familiarity and a wide use of all their functionalities. To promote the successful deployment of new information systems in an organization, it is essential to evaluate from its initial conception, the conditions for an effective implementation.^2^^,^^3^ This evaluation should enable decision-makers to analyze the factors promoting or hindering the acceptability of the digitalization of EMRs by health professionals.

Several studies highlight factors influencing the use of EMRs after their implementation in healthcare facilities,^4^^,^^5^ with an emphasis most of the time, on the actual use of the EMR by health professionals.^6–9^ In contrast, the study performed here proposes to evaluate and identify determinants of the intention to use the EMR before the completion of the deployment of the system with the aim to develop strategies that foster conditions suitable for its use.

## THEORETICAL MODEL

The technology acceptance model (TAM) developed by Davis^8^ seems to be often used to study the routine utilization of new information technologies.^10^ This model was designed to explain the behavior of users of information and communication technologies (ICTs) and the perceived performance of these ICTs. The TAM is based on 2 theories: the theory of reasoned action (motivation and usage ability) and the theory of expectations (perceived consequences).

Most studies assessing the acceptability of information technology in healthcare systems and more specifically, the acceptance of EMR, have used the TAM model. It proposes 2 main factors determining the use of new technologies: perceived usefulness and perceived ease of use.^8^ The TAM takes also into account external variables, such as experience, motivation, and subjective norms, which can indirectly determine the acceptance of new technologies.^8^

The TAM provides a relevant framework for analyzing perceptions of users of new technologies in health systems.^4^^,^^11^^,^^12^ In the study carried out by Gagnon and collaborators on the acceptance of the digital health record by physicians, the TAM was chosen to predict the intention of physicians to use the EMR.^13^ The TAM was also used in assessing the satisfaction of users with a CIS and in identifying determinants of overall satisfaction for that CIS.^6^ Several other researchers have used the TAM in health to predict the intention of health professionals to use new information technologies for the management of patient care.^14–19^

**Figure 1.**
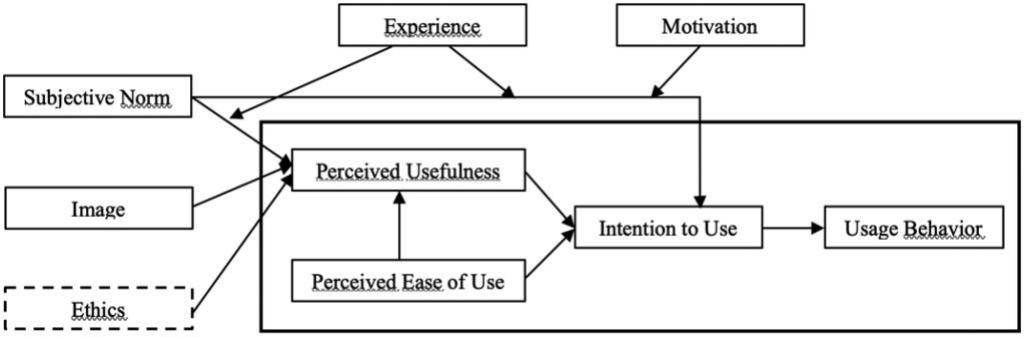
Study design inspired by TAM 2.

In this study, the original TAM 2 was modified. Indeed, dimensions such as “job relevance,” “output quality,” and “result demonstrability” were not retained in the empirical analysis since the study performed in this case is upstream of the actual use of the EMR. Furthermore, the ethical dimension was added to the model chosen for this study because it seemed to be a better approach for a good assessment of potential obstacles to the intention of health professionals to use the EMR (Figure 1).

Depending on the model chosen, the perceived usefulness and the perceived ease of use jointly explain the intention of health professionals to use the EMR. Perceived usefulness is the degree to which a person thinks that using a system can improve their professional performance. Perceived ease of use, on the other hand, is the degree to which a person thinks that the use of a system would not need too much effort.

The perceived usefulness is justified by subjective norms such as image, experience, motivation, and ethical dimensions that indirectly explain the intention of health professionals to use the EMR.

The subjective standard refers to the influence exerted by colleagues on the acceptance of the EMR. The image is used to qualify the perception of users on different features of the EMR. By ethics, we mean the ability of the EMR to respect medical confidentiality. Experience refers to the previous use of the EMR while motivation is associated with anything that leads to the use of the instrument.

## DATA AND METHOD

A questionnaire was constructed based on the theoretical model TAM 2 chosen for this study. It was inspired by the questionnaire used in the work performed by Sié Palm^20^ on the factors of acceptability of the CIS by health professionals. Its design considered the fact that the use of the EMR in health facilities in Gabon is relatively new, which guided the choice for pre-use evaluation. Not all variables that could measure actual use in the work of Palm were retained for this study. Only dimensions related to the intention to use the EMR by health professionals were retained.

Eight dimensions were selected in this questionnaire, namely: socio-demographic characteristics (including experience of computer use and experience of using the EMR), perceived ease of use of the EMR, perceived usefulness, motivation for use, subjective standard, intention to use, ethics related to the use, and functional image of the EMR (see the questionnaire in the Supplementary Annex). Each dimension was assessed through a set of questions evaluated on a 5-level Likert scale.

The questionnaire was administered through a face-to-face interview with health professionals in their respective workplaces. It was administered comprehensively to health professionals of university hospitals, hospitals, polyclinics, and clinics in Libreville, Gabon. As the investigation team conducted the survey, no refusals were reported. Nevertheless, the only healthcare workers who did not take the survey are those who were involved in the fieldwork for the management of COVID-19. Data were collected from September 21 through October 13, 2020.

Analyses were carried out using the statistical software R. For all analyses, the significance threshold was set at .05.

## RESULTS

A total of 218 health professionals participated in the study conducted. Twenty-eight percent (28%) of respondents were male. The average age of participants was 41.3 years (±8.9 years) with 12.02 years (±8.47 years) of work experience in the healthcare industry. Almost 60% (59.6%) of respondents indicated their intention to use the EMR in their professional routine. Table 1 presents the socio-demographic characteristics of surveyed participants.

**Table 1.** Socio-demographic characteristics of professionals

Characteristics	Number (*N* = 218)	Percentage
Sex		
Female	157	72.0
Male	61	28.0
Profession		
Biologist (aka, laboratory worker)	20	9.2
Nurse	95	43.6
Physician	32	14.7
Radiologist	6	2.8
Midwife	60	27.5
Medical secretary	3	1.4
Physiotherapy technician	2	0.9
Age group		
≤30	28	12.8
[30–40]	75	34.4
[40–50]	82	37.6
>50	33	15.1
Seniority		
≤10	103	48.6
[10–20]	83	39.2
[20–30]	21	9.9
[30–40]	5	2.4
EMR		
No	172	78.9
Yes	46	21.1

A variable was constructed for each dimension of the theoretical model. The variables were obtained by adding the Likert scale scores of the applied questionnaire. Table 2 shows all the dimensions used for the construction of the analysis variables. The validity of each dimension was tested using alpha coefficients of Cronbach. The values in this test were all greater than or equal to 0.7. According to the literature, values greater than or equal to 0.6 were considered acceptable.^6^^,^^15^^,^^16^ A Cronbach’s alpha coefficient greater than 0.80 is considered very good.

**Table 2.** Construction of variables (alpha of Cronbach)

Variables	Average	Standard deviation	Alpha of Cronbach
Experience using the computer	2.57	0.87	
Your level of proficiency in computer use in general	2.19	1.04	0.76
Your level of proficiency in text processing (MS Word)	2.44	1.04	
Your level of proficiency in sending electronic messaging	2.86	1.26	
Your level of mastery of digital research of clinical information	2.80	1.19	
Perceived ease of use of the EMR	3.01	0.55	0.72
Access to the EMR is simple	2.94	0.65	
Using the EMR is easy and ergonomic	3.00	0.7	
When I encountered a problem, I received the appropriate help	3.06	0.69	
Perceived usefulness of using the EMR	4.10	0.62	0.84
Using an EMR allows you to better track your patients	4.20	0.91	
Using an EMR allows you to view laboratory results	4.32	0.94	
Using an EMR allows you to view imaging results	2.72	0.89	
Using the EMR allows me to improve my efficiency in my practice	4.33	0.94	
Using the EMR improves my decision-making	4.20	1.01	
Using the EMR allows me to always have an up-to-date and synthetic medical history, including the elements useful for decision-making	4.42	0.86	
Using the EMR allows documenting the history of acts and prescriptions related to the management of patients	4.36	0.93	
Using the EMR allows sharing of laboratory test results and radiology examinations	4.26	1.02	
Using the EMR allows the sharing of information between physicians for continuity of care	4.45	0.84	
Using the EMR allows information to be shared with nurses for the development of a care plan	4.30	0.94	
Using the EMR allows the review of medical data by the patient	3.54	1.48	
Motivation for the use of the EMR	3.68	1.04	0.81
Using the EMR meets my work habits	3.55	1.14	
Using the EMR perfectly meets the organization of my work	3.81	1.12	
Ethics related to the use of an EMR	4.34	0.89	0.70
The EMR must guarantee medical confidentiality	4.41	1.01	
The EMR must guarantee the proper security of health information	4.26	1.02	
Subjective norm			0.95
I will use it if my colleagues do	3.26	1.46	
EMR experience			
Your level of knowledge on EMR	2.57	0.82	
Intent to use the EMR			
I intend to use it	4.34	0.98	
Functional image	3.89	0.82	
Admission, departure, and transmission	3.80	0.99	
Scheduling appointments	4.01	1.01	
Writing the discharge sheet	3.85	0.96	
Writing the hospitalization sheet	3.87	0.92	
Entry of medical prescriptions	3.94	1.01	
Entry of nursing transmissions	3.83	1.01	
Visualization of nursing transmissions	3.83	1.01	
Visualization of the imaging report	3.91	0.98	
Visualization of care plans	3.93	0.95	
Visualization of medical imaging	3.84	1.01	
Visualization of medical biology results	4.00	1.01	

The purpose of the empirical analysis was to identify the determinants of intent to use the EMR tool. The dependent variable “intention to use” was a recoded binary variable grouping the terms “strongly disagree,” “disagree,” and “do not know” on the one hand, “somewhat agree” and “strongly agree” on the other hand. A second recoding on the same principle was carried out for the perceived usefulness variable. Of the total sample, 59.6% of respondents strongly agreed on their intention to use the EMR, 24.8% somewhat agreed, 8.3% had no idea about their intention to use the EMR; 7.3% did not intend to use the EMR in their medical practice.

Three regression models were implemented: (i) a multivariate model explaining the intention to use (Logit) by all the dimensions of the theoretical model, (ii) a multivariate model that explains the perceived usefulness of the relevant dimensions about the TAM model, and finally, (iii) a regression tree in order to identify typical profiles of health professionals reporting not intending to use the EMR.

Multivariate regression (Logit) highlights a significant link between the intention of health professionals to use the EMR and the perceived usefulness variable on the one hand and the subjective norm on the other hand. The intention of professionals to use the EMR is explained by the perceived usefulness of professionals (*P* = .0005) and the subjective standard (*P* = .01).

No socio-demographic variable explains the intention to use the EMR by health professionals (Table 3) which highlights the central role of mediating variable of the “perceived usefulness” dimension as suggested by the TAM.

**Table 3.** Multivariate models explaining intent to use

Variables	OR	95% CI	*P*-value
Perceived usefulness	5.3264	[2.2375–14.7250]	.0005
Subjective norm	1.8098	[1.1376–3.0009]	.0157

Table 4 explains the perceived usefulness of the other variables and tests the validity of our TAM. In accordance with the TAM theoretical model, results show a significant effect of “motivation” (*P* = .0021), “subjective norm” (*P* = .003), image that health professionals have regarding the EMR (*P* = .008), and perceived “ethics” (*P* = .0005).

**Table 4.** Multivariate model explaining the perceived usefulness

Variables	OR	95% CI	*P*-value
Profession (physician)	0.2318	[0.0418–1.1631]	.0796
Sex (male)	3.7002	[0.9268–16.9482]	.0743
Age	1.1054	[1.0364–1.1868]	.0035
Motivation	2.2650	[1.3693–3.9140]	.0021
Subjective norm	2.1715	[1.3391–3.7682]	.0030
Image	2.3592	[1.2832–4.6292]	.0080
Ethics	2.3419	[1.4641–3.8612]	.0005

Socio-demographic characteristics of the study population were not correlated with the perceived usefulness of the EMR by health professionals, except for age (*P* = .0035).

**Figure 2.**
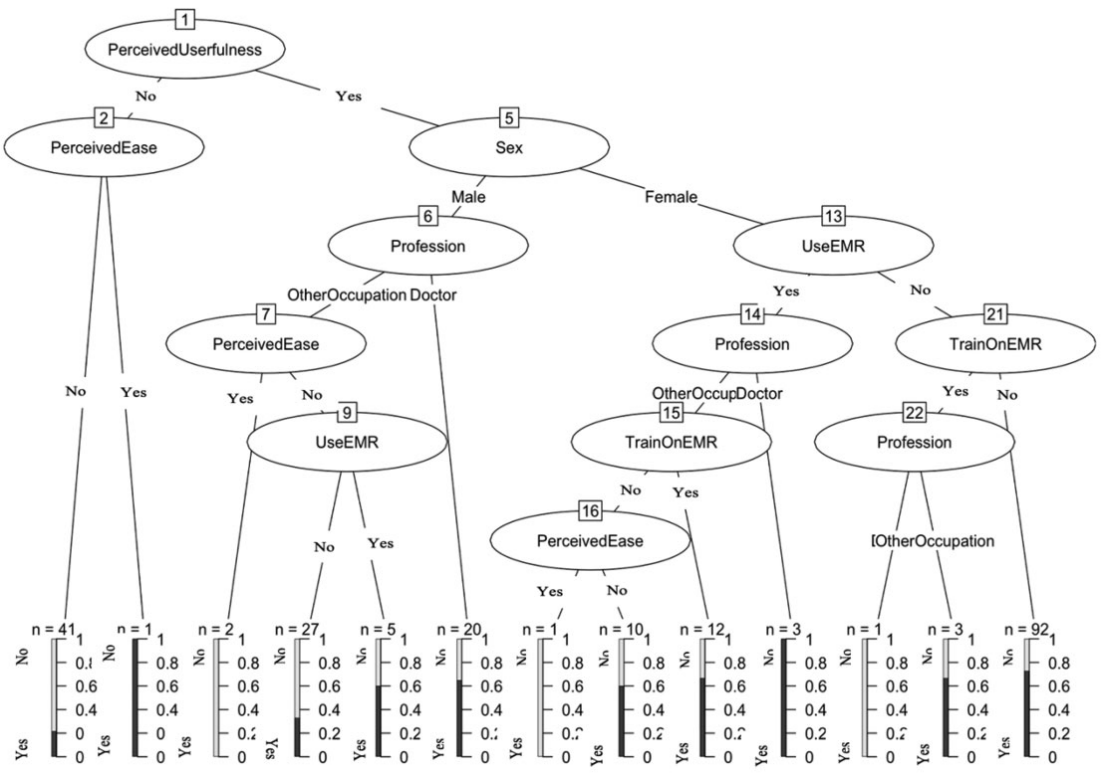
Regression tree (variable of interest “intention to use the EMR”). *The base of the decision tree represents the intention of the professionals to use the EMR

A decision tree was created to present the distribution of health professionals on the intention to use the EMR in their professional practice for the management of patients (Figure 2). As predicted by the TAM 2 model, the most discriminating factor is perceived usefulness. Out of the 42 health professionals who did not perceive the usefulness of the EMR, 41 had a negative perception of the ease of use of this tool. Furthermore, 40 did not intend to use the EMR. In this subpopulation, no other discriminating factors were identified. Thus, the model identified a population of health professionals reluctant to use the EMR without any other factor to characterize it, which does not give immediate leverage for action to promote their adherence. In contrast, among those who perceived the usefulness of the EMR, the majority of those who did not anticipate using the tool had a negative perception of ease of use. Among these, 92 had not received training on the use of the EMR. The decision tree here made it possible to identify a simple action lever to promote the intention to use the tool.

## DISCUSSION

The aim of the study was to assess the intention of health professionals to use the EMR in healthcare facilities in Libreville and Owendo in Gabon. TAM 2 was adopted as the analytical model and the dependent variable in this study was the intention to use the EMR. The results of the analysis performed suggest that the intention of health professionals to use the EMR is significantly associated with perceived usefulness and subjective norm. The subjective norm was the social influence exerted by society on health professionals regarding the use of this EMR. However, these results were not fully consistent with those observed in some studies that have addressed the same issue.^14^^,^^21–25^ In these studies, the outcome similar to ours was perceived usefulness, which explained healthcare professionals’ intention to use the EMR. The second variable in these studies that explained healthcare professionals’ intention to use the EMR was perceived ease of use of the EMR. This result is not similar to the one obtained in our study, which presents subjective norm as the second variable explaining healthcare professionals’ intention to use the EMR in their professional practice.

The perceived usefulness associated with the intention to use the tool was found in studies done by Chau and Hu^26^ and Walter and Lopez.^27^ These studies, while assessing the intention of physicians to use the electronic health record, show the same results as the one presented here. This dimension was the most important factor influencing the intention to use information technology in the field of health.

The study performed here reveals the subjective norm as the second factor explaining the intention of health professionals to use the EMR. This dimension was the third in the study of Gagnon et al.^13^ Although health professionals are autonomous in their practice, peer influence could strongly affect the decision of the staff in the acceptance of EMRs and also on the use of the latter.

Perceived ease of use has been significantly associated with the intention to use health information technology found in some studies.^13^^,^^24^^,^^25^ This finding is contrary to that of the study reported here, which found no link between perceived ease of use and intention to use the EMR. This observation could be explained by the fact that there are few users of the EMR in the population sample used in this study.

The same finding was made for experience and ethics that were not associated with the intention to use the EMR in this study, unlike Choi et al.’s.^23^ The ethical dimension was used in order to protect patient privacy. The remaining variables in the model used in this study were also unassociated with the intention of health professionals to use the EMR. This is the case of the knowledge of the computer tool, motivation, and the image health professionals have of the EMR in daily tasks and which was integrated as a component into this analysis. These results are different from those reported by Gagnon et al,^13^ which highlighted the knowledge of information technologies and the use of computers in the field of health. This was also proven in the study performed by Venkatesh et al,^28^ which stated that health personnel who were able to use information technology had fewer difficulties using the EMR. One of the constraining factors in relation to the use of the EMR is the perceived difficulty of using the IT which is part of the EMR. Mezni et al^29^ had referred to this difficulty in handling the EMR as a constraining factor for users. Computer training is the only way to facilitate the use of the computer tool or the EMR. More than half of the respondents in this study had no training for the use of the computer tool. This weakness in training could explain the difficulty of use perceived by the health professionals who responded to the survey in the work performed here.

In terms of socio-demographic characteristics, there were no significant links between the intention to use and these characteristics. The same observation was also made on perceived usefulness, except for age. A result that does not seem to align with the results of other studies found in the literature.^27^^,^^28^^,^^30^ Like this study, that of Gagnon et al, highlights the age that would influence the perceived usefulness for health professionals. Indeed, physicians under the age of 50 found the EMR to be very useful compared with others. In their study (Gagnon et al), sex also was associated with the perceived usefulness as opposed to the results shown here. For that research team, the influence of the subjective norm on the intention to use was more important for females than males. Walter and Lopez^27^ also reported a significant difference between sex for the intention to use the EMR. Sex is presented as a factor acting on the social influence of colleagues regarding the use of EMR.

An ethical dimension was included in this report to give more precision or explanations on the motivation of health professionals on their intention to use the EMR in their medical practice. The ethical dimension here refers to the protection of personal data of patients and the guaranteed medical confidentiality, which could constitute an obstacle to the use of the EMR. Although this dimension was strongly associated with the perceived usefulness of the EMR, motivation was not associated with the intention to use the EMR. The choice to integrate this dimension was done based on the observation of the conservative nature of health professionals in terms of health data in services. Patient data are typically kept by prescribing physicians and are not shared for patient follow-up outside the service. One of the reasons for this refusal to share is related to medical confidentiality.

The present study is not without limitations. Health professionals were poorly represented due to their unavailability during the visit of interviewers. This unavailability could be explained by the deployment of these professionals to the many COVID-19 management units developed across the nation in response to the rise of the pandemic.

## CONCLUSION

The intention to use the EMR was strongly associated with the perceived usefulness and subjective norm of health professionals. Using the EMR did not appear to be easy for much of the population sample surveyed. This difficulty of use presented by health professionals could be explained by the lack of training in the use of the EMR in healthcare facilities. The perceived ease of use and lack of training are the potential obstacles to the use of the EMR. Training on IT tools and on the use of the EMR must be given to health professionals to hope for the use of IT devices in the field of health. This training should be integrated into the initial training curricula in schools and universities, and in continuing education for professionals already in full-time practice. In addition to training, awareness-raising actions on the importance of the EMR are necessary for its acceptance by future users.

## Supplementary Material

ooac096_Supplementary_Data

## Data Availability

The data underlying this article will be shared on reasonable request to the corresponding author.
