# Supplementary material for: Evaluation of the intention to use the electronic medical record (EMR) by health professionals in healthcare facilities of Libreville and Owendo in Gabon
Source: JAMIA Open. 2022 Nov 22;5(4):ooac096. doi: 10.1093/jamiaopen/ooac096 (PMC9680664; doi:10.1093/jamiaopen/ooac096)
Supplement: ooac096_Supplementary_Data [file ooac096_supplementary_data.docx]

Lay Summary

Health systems in several countries have opted to implement electronic health records (EMRs) to track patients throughout their care. The objective behind these EMRs is to allow for the exchange of patient information between physicians and/or between care facilities for continuity of care. The operation of this system requires that it be accepted by the health care staff who are the primary users. This study assesses the intention of health professionals to use the EMR in the management of the Gabonese patient. A questionnaire was administered to health professionals to measure their intention to use the EMR.

Intention to use the EMR was based on perceived usefulness of the EMR, subjective norm (influence of colleagues) and experience with the use of IT devices in patient care.

However, we regret the lack of staff training in the use of computers in medical practice. This is a real obstacle to the use of EMRs in health care facilities.
